# Supplementary material for: PTCHD1 Binds Cholesterol but Not Sonic Hedgehog, Suggesting a Distinct Cellular Function
Source: Int J Mol Sci. 2023 Jan 31;24(3):2682. doi: 10.3390/ijms24032682 (PMC9917202; doi:10.3390/ijms24032682)
Supplement: Supplementary file 1 [file ijms-24-02682-s001.zip › 2022_IJMS_PTCHD1_supplementary.pdf]

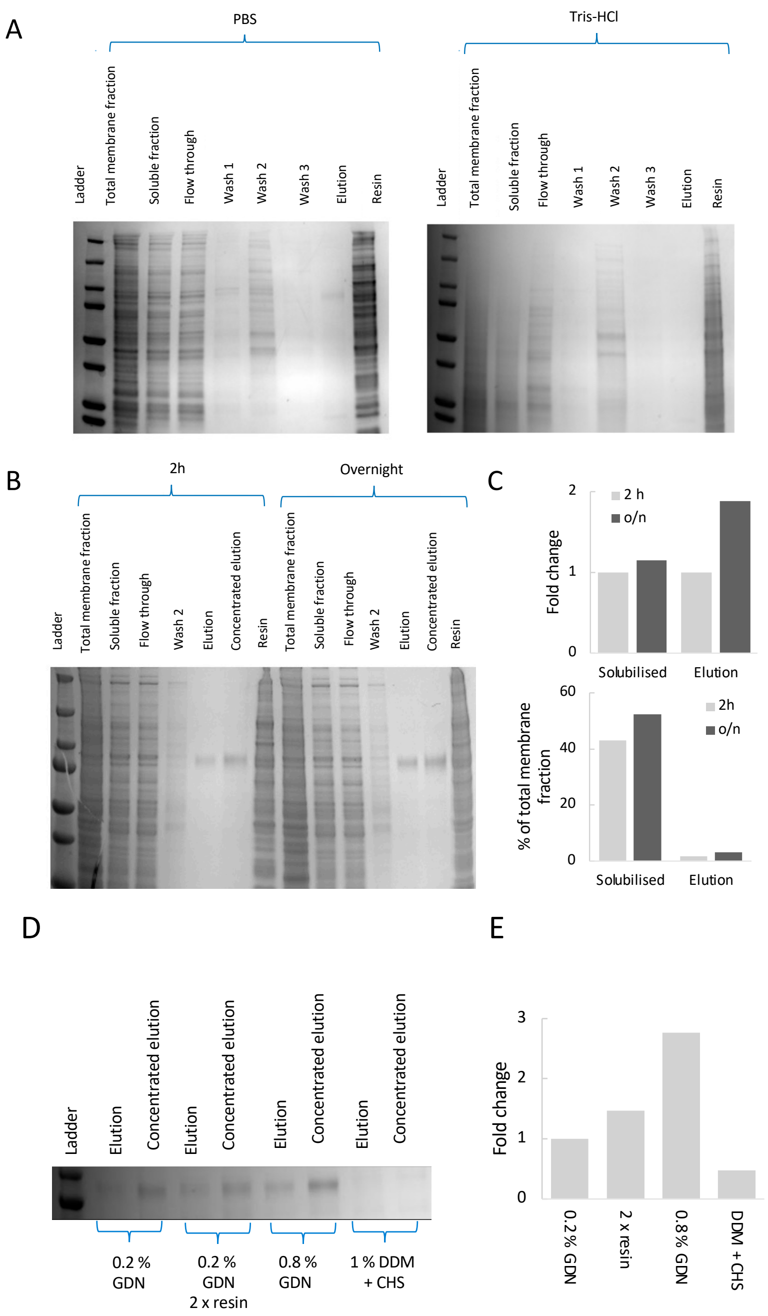

S1 Optimization of PTCHD1 purification. A Purification of PTCHD1 in either PBS or Tris-HCl. A clear band is visible in the elution fraction of the PBS purification, whereas no band is visible in the Tris-HCl elution fraction. **B** PTCHD1 purification following solubilization in 0.2 % GDN for either 2 h or overnight at + 4 C **C** Densitometry analysis shows a modest increase in o/n solubilization compared to 2 h solubilization (1.15 -fold increase), and a 1.9 -fold increase in eluted PTCHD1. **D** Clear differences in the intensity of PTCHD1 elution bands (~100 kDa) after altering the resin amount, detergent amount or detergent type. **E** Densitometry analysis shows a 1.5 -fold increase in eluted PTCHD1 when the resin amount was doubled, and 2.7 -fold increase when the amount of GDN used to solubilize PTCHD1 was increased to 0.8 %. Using 1 % DDM supplemented with 0.125 % CHS led to a decrease in eluted PTCHD1.

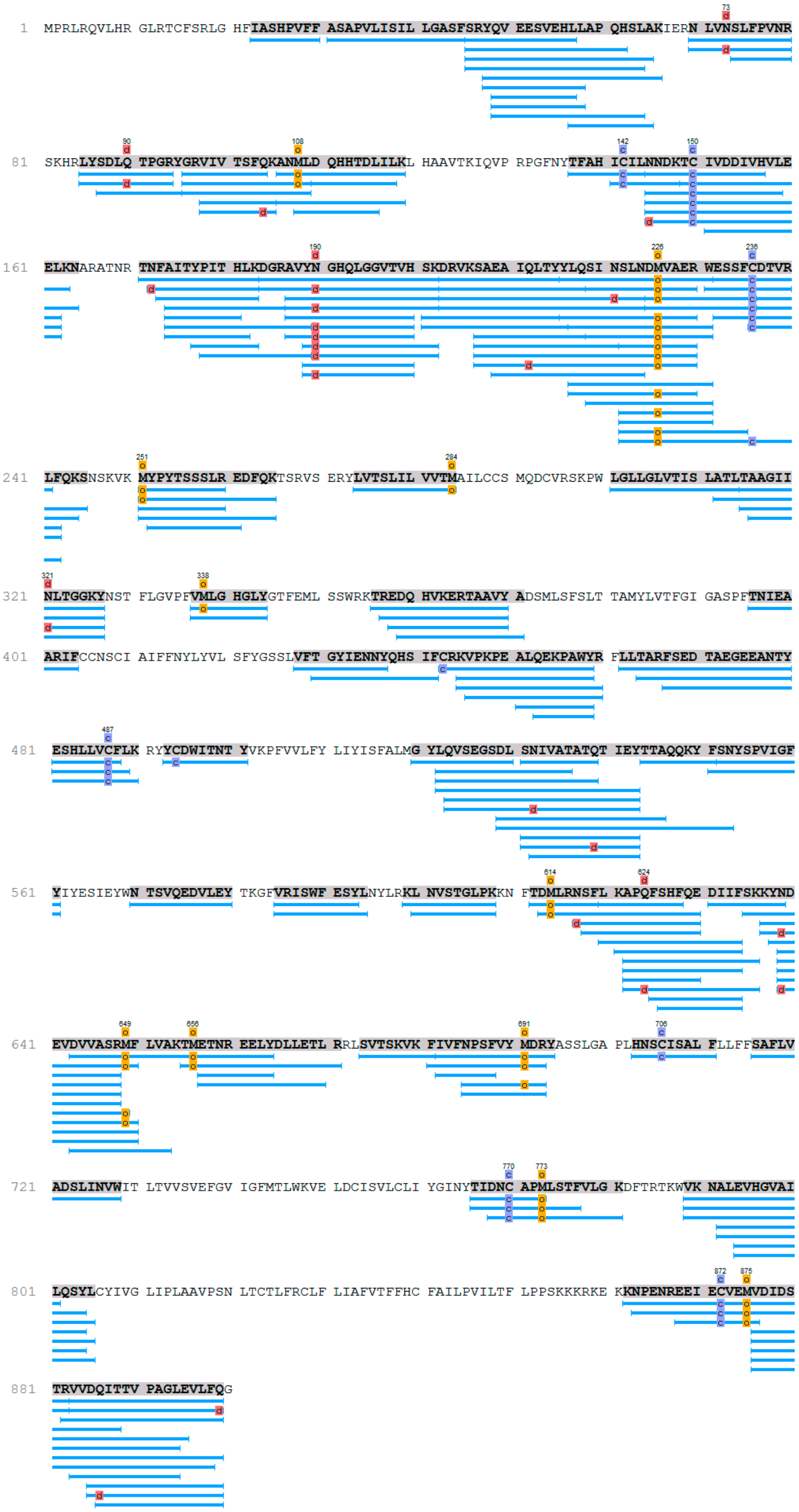

S2 Mass spectrometry sequence coverage of purified PTCHD1.

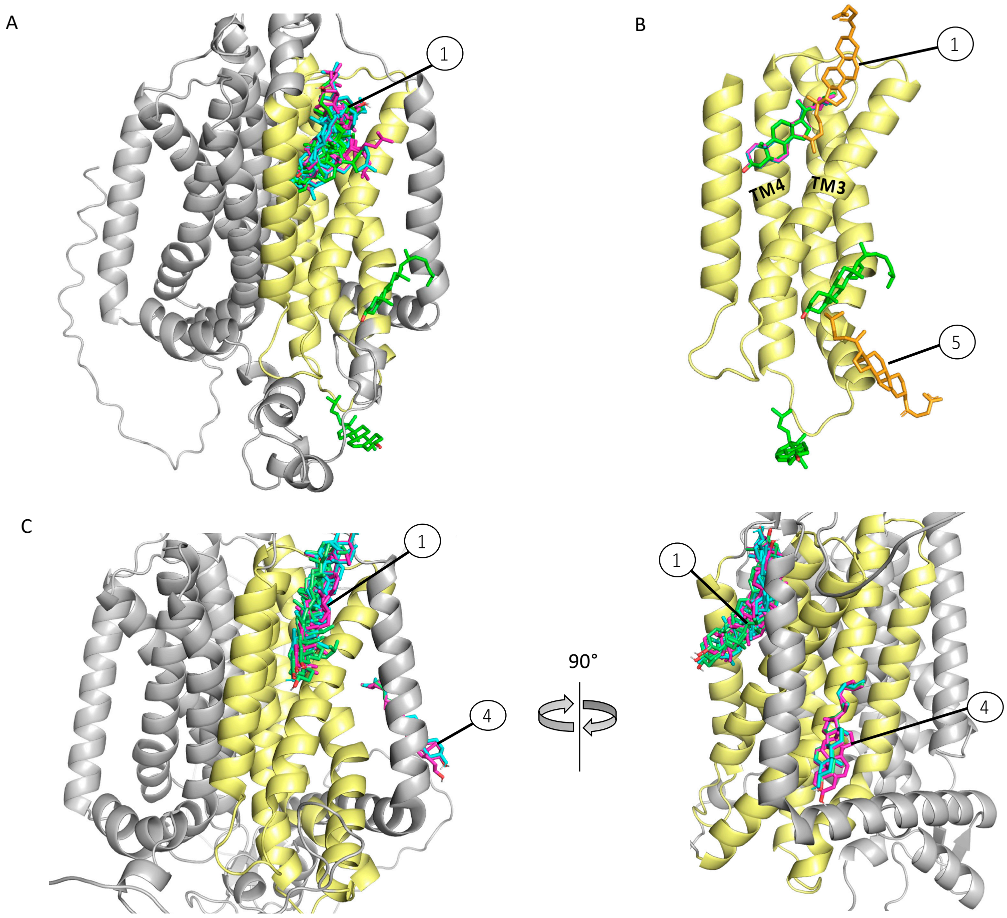

S3 Docking of cholesterol to PTCHD1 and PTCHD1 indicates similar binding. Docking was performed in triplicates, cholesterols from the first, second, and third runs are in cyan, lime, and pink, respectively. Sites are numbered as in Qi et al. (2018)[28] **A** All cholesterols docked to PTCHD1. 25/27 cholesterols were docked to a position corresponding to site 1. **B** The cholesterol with highest affinity to PTCHD1 (lime, top) overlaps with PTCH1 (6RMG) cholesterol site 1 (orange, top). Two outlying cholesterols were docked near PTCH1 cholesterol position 5 (orange, bottom) **C** Cholesterols dock to two identified sites on PTCH1 (AF-Q13635). Sites 1 and 4 are occupied by 23 and 4 cholesterols, respectively.

Supplementary table 1 Docking results

TARGET	RUN	MODE	AFFINITY (KCAL/MOL)	SITE	TARGET	RUN	MODE	AFFINITY (KCAL/MOL)	SITE
**PTCHD1**	1	1	-8,6	1	**PTCH1**	1	1	-8,4	4
**PTCHD1**	1	2	-8,6	1	**PTCH1**	1	2	-8,3	1
**PTCHD1**	1	3	-8,0	1	**PTCH1**	1	3	-8,1	1
**PTCHD1**	1	4	-7,7	1	**PTCH1**	1	4	-8,1	1
**PTCHD1**	1	5	-7,7	1	**PTCH1**	1	5	-8,0	1
**PTCHD1**	1	6	-7,7	1	**PTCH1**	1	6	-8,0	1
**PTCHD1**	1	7	-7,6	1	**PTCH1**	1	7	-7,6	1
**PTCHD1**	1	8	-7,5	1	**PTCH1**	1	8	-7,6	1
**PTCHD1**	1	9	-7,4	1	**PTCH1**	1	9	-7,6	1
**PTCHD1**	2	1	-8,6	1	**PTCH1**	2	1	-8,3	4
**PTCHD1**	2	2	-8,0	1	**PTCH1**	2	2	-8,2	1
**PTCHD1**	2	3	-7,6	1	**PTCH1**	2	3	-8,0	1
**PTCHD1**	2	4	-7,5	1	**PTCH1**	2	4	-7,9	1
**PTCHD1**	2	5	-7,5	1	**PTCH1**	2	5	-7,7	1
**PTCHD1**	2	6	-7,4	1	**PTCH1**	2	6	-7,7	1
**PTCHD1**	2	7	-7,4	1	**PTCH1**	2	7	-7,6	1
**PTCHD1**	2	8	-7,2	5	**PTCH1**	2	8	-7,5	1
**PTCHD1**	2	9	-7,2	5	**PTCH1**	2	9	-7,3	1
**PTCHD1**	3	1	-8,7	1	**PTCH1**	3	1	-8,1	4
**PTCHD1**	3	2	-8,0	1	**PTCH1**	3	2	-8,1	1
**PTCHD1**	3	3	-7,7	1	**PTCH1**	3	3	-7,8	1
**PTCHD1**	3	4	-7,7	1	**PTCH1**	3	4	-7,7	1
**PTCHD1**	3	5	-7,7	1	**PTCH1**	3	5	-7,6	1
**PTCHD1**	3	6	-7,5	1	**PTCH1**	3	6	-7,5	1
**PTCHD1**	3	7	-7,5	1	**PTCH1**	3	7	-7,4	1
**PTCHD1**	3	8	-7,5	1	**PTCH1**	3	8	-7,4	1
**PTCHD1**	3	9	-7,3	1	**PTCH1**	3	9	-7,3	4

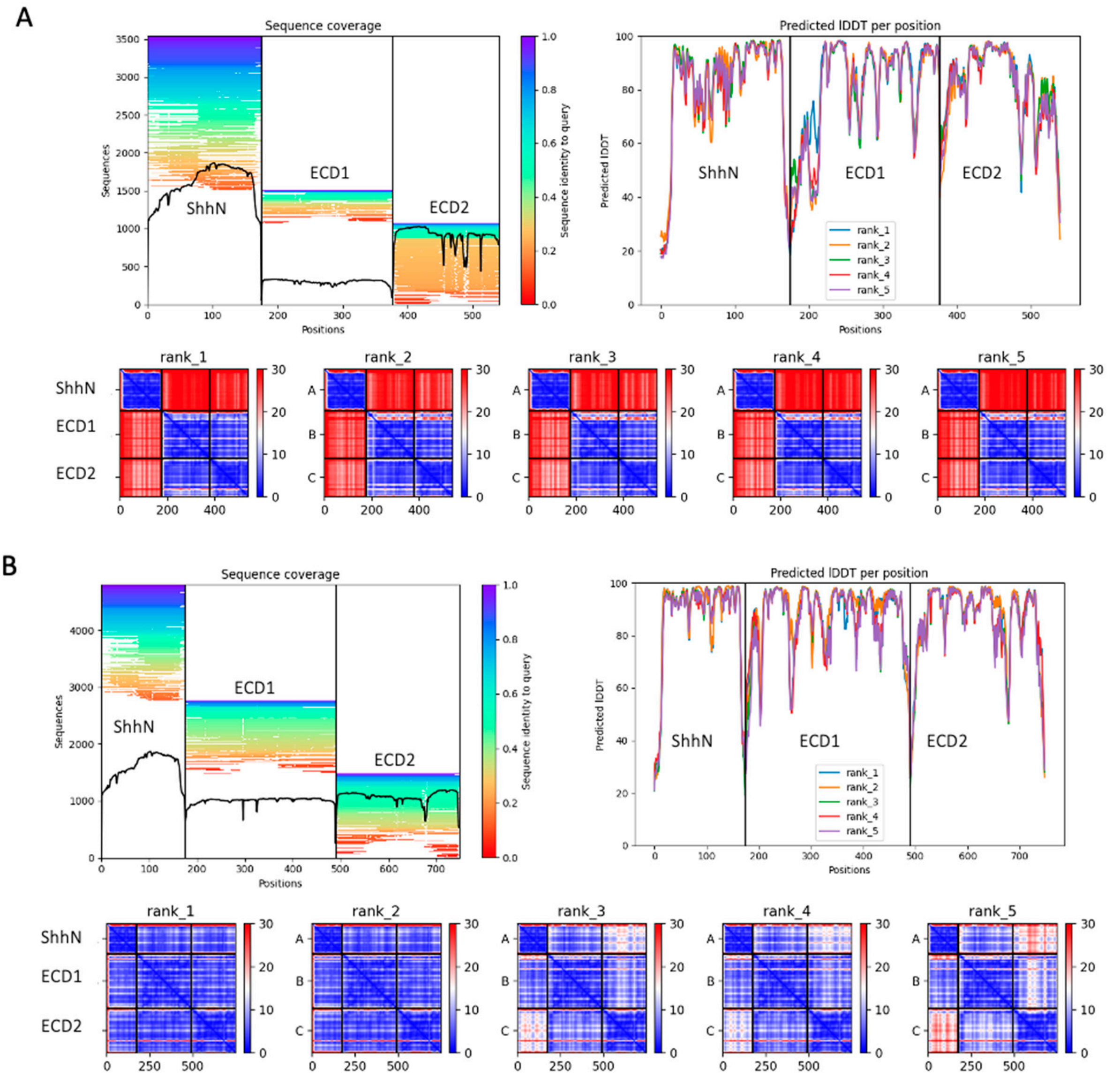

S4 AlphaFold2 prediction of ECD1:ECD2:ShhN complex formation. (A) PTCHD1 ECDs do not form a complex with ShhN. The sequence coverage is acceptable throughout and the predicted local distance difference test (lDDT) score is above 50 excluding N-terminals of the ShhN and ECD1, and C-terminal of ECD2. The predicted aligned error is high in all ranks between both ECDs and ShhN. **(B)** PTCH1 ECDs form a complex with ShhN. The sequence coverage is acceptable throughout and the predicted lDDT is above 50 excluding N-terminals of the ShhN and ECD1, and C-terminal of ECD2. The predicted aligned error is low throughout in ranks 1 and 2, and in most positions of rank 4. In rank 5 the predicted aligned error is high between ShhN and ECD2.

```
Consensus              MXXXXXXXXXXXXXXXXXX XXXXXXXXXXXXXXXXXXX XXXXXXRXXLHRXXXXXX     58
Q13635|PTCH1_HUMAN     MASAGNAAEPQDRGGGGSGC IGAPGRPAGGGRRRRTGGLR RAAAPDRDYLHRPSYCDA   58
Q96NR3|PTCHD1_HUMAN    ML------------------ -------------------- ------RQVLHRG-----    9

Consensus              XX XXXXXXXXXXXXXXXXLR XXXXXXXXXLGXXIXXXXXX FXXXXXLIXXXXXXXX  116
Q13635|PTCH1_HUMAN     AF ALEQISKGKATGRKAPLWLR AKFQRLLFKLGCYIQKNCGK FLVVGLLIFGAFAVGL  116
Q96NR3|PTCHD1_HUMAN    -- ------------------LR TC----FSRLGHFIASHPVF FASAPVLISILLGASF   43

Consensus              XXXX XEXXVEXLXXXXXXXXXXEX NXXXXXXXXXXXXXXXXXXJ QTPXXXGXXXXTXX  174
Q13635|PTCH1_HUMAN     KAAN LETNVEELWVEVGGRVSREL NYTRQKIG-EEAMFNPQLMI QTPKEEGANVLTTE  173
Q96NR3|PTCHD1_HUMAN    SRYQ VEESVEHLLAPQHSLAKIER NLVNSLFPVNRSKHRLYSDL QTPGRYGRVIVTSF  101

Consensus              XXXXXL DXXXXXXXLXXXXXXXXXXV XXXXXXXXXXHJCXXXXXXX XXTXXXDXIXXX  232
Q13635|PTCH1_HUMAN     ALLQHL DSA-----L----QASRVHV YMYNRQWKLEHLCYKSGELI TETGYMDQIIEY  222
Q96NR3|PTCHD1_HUMAN    QKANML DQHHTDLILKLHAAVTKIQV PRPGFNYTFAHICILNND-- -KTCIVDDIVH-  155

Consensus              XXXXXXXX XXXXXXXXXXXXXXXXXXXX XXXXXXXXXXXXXLEELKX XXXXXXXXXX  290
Q13635|PTCH1_HUMAN     LYPCLIIT PLDCFWEGAKLQSGTAYLLG KPPLRWTNFDPLEFLEELKK INYQVDSWEE  280
Q96NR3|PTCHD1_HUMAN    -------- -------------------- -------------VLEELKN AR--------  164

Consensus              XXXXXXXXXX XXXXXXXXXXXXXXXXXAXN XXXXXXXXXXTXXXDXXXVX NGXXXXXX  348
Q13635|PTCH1_HUMAN     MLNKAEVGHG YMDRPCLNPADPDCPATAPN KN-------STKPLDMALVL NGGCHGLS  331
Q96NR3|PTCHD1_HUMAN    ---------- -----------------ATN RTNFAITYPITHLKDGRAVY NG------  189

Consensus              XXXXXXXXXXXX GGXXXXSXXXXXSAXAJQXX XXLXXXXXXXXXXXXXXXXX XXXXXX  406
Q13635|PTCH1_HUMAN     RKYMHWQEELIV GGTVKNSTGKLVSAHALQTM FQLMTPKQMYEHFKGYEYVS HINWNE  389
Q96NR3|PTCHD1_HUMAN    ---------HQL GGVTVHSKDRVKSAEAIQLT YYLQSINSLNDM-------- ------  224

Consensus              XXXXXXXEXWZXXX XXXVXXXXXXXNSXXKXXXXXT XXXLXXXXXXXSXVSXXXXX XXXJ  464
Q13635|PTCH1_HUMAN     DKAAAILEAWQRTY VEVVHQSVAQNSTQKVLSFT TTTLDDILKSFSDVSVIRVA SGYL  447
Q96NR3|PTCHD1_HUMAN    -----VAERWESSF CDTVRLFQKSNSKVKMYPYT SSSLREDFQKTSRVSERYLV TSLI  277

Consensus              LXXXXAXLXXXXXDCX XSXXXXGLXGXXXXXLXXXX XXGJXXLXGXXXNXXXXXXX PF  522
Q13635|PTCH1_HUMAN     LMLAYACLTMLRWDCS KSQGAVGLAGVLLVALSVAA GLGLCSLIGISFNAATTQVL PF  505
Q96NR3|PTCHD1_HUMAN    LVVTMAILCCSMQDCV RSKPWLGLLGLVTISLATLT AAGIINLTGGKYNSTF-LGV PF  334

Consensus              XXLGXGXXXXFXXXXXXX XTXZBXXXXXXXRTXXXXXX XXXSXXLTXXXXXXFXXXA  580
Q13635|PTCH1_HUMAN     LALGVGVDDVFLLAHAFS ETGQNKRIPFEDRTGECLKR TGASVALTSISNVTAFFMAA  563
Q96NR3|PTCHD1_HUMAN    VMLGHGLYGTFEMLSSWR KTREDQHV--KERTAAVYAD SMLSFSLTTAMYLVTFGIGA  390

Consensus              XXXXXIXAXRXFXXXXXXXX XFNXXXVLXXXXXXLXXXXY XXXBXXXXIFCXXXXPXX  638
Q13635|PTCH1_HUMAN     --LIPIPALRAFSLQAAVVV VFNFAMVLLIFPAILSMDLY RREDRRLDIFCCFTSPCV  619
Q96NR3|PTCHD1_HUMAN    SPFTNIEAARIFCCNSCIAI FFNYLYVLSFYGSSLVFTGY IENNYQHSIFCRKV-P-K  446

Consensus              XX XJQXXPXXYXXXXXXXXXXX XXXXXXXXXXXXXXXXXXXX XXXXXXXXXXXXXXXX  696
Q13635|PTCH1_HUMAN     SR VIQVEPQAYTDTHDNTRYSP PPPYSSHSFAHETQITMQST VQLRTEYDPHTHVYYT  677
Q96NR3|PTCHD1_HUMAN    PE ALQEKPAWYRF--------- -------------------- --------------LL  461

Consensus              TXXX XXXXXXXXXXXXXXXXXXXX XXXXXXXXXXXXXFSXXXXX XXEXXXXXXXXLXX  754
Q13635|PTCH1_HUMAN     TAEP RSEISVQPVTVTQDTLSCQS PESTSSTRDLLSQFSDSSLH CLEPPC-TKWTLSS  734
Q96NR3|PTCHD1_HUMAN    T--- -------------------- -----------ARFSEDTAE GEEANTYESHLLVC  485

Consensus              FXXXXY XXXJXXXXXKXXVXXXXLXX JXXXLXGXXXVXXGXDLXBI VXXXTXXXXXXX  812
Q13635|PTCH1_HUMAN     FAEKHY APFLLKPKAKVVVIFLFLGL LGVSLYGTTRVRDGLDLTDI VPRETREYDFIA  792
Q96NR3|PTCHD1_HUMAN    FLKRYY CDWITNTYVKPFVVLFYLIY ISFALMGYLQVSEGSDLSNI VATATQTIEYTT  543

Consensus              AQXKYFSX YXXXIXXXXXZXXXYXNXXX XXXXXXXXXXXVXXXXLEXX KXXXXXXWXX  870
Q13635|PTCH1_HUMAN     AQFKYFSF YNMYI---VTQKADYPNIQH LLYDLHRSFSNVKYVMLEEN KQLPK-MWLH  846
Q96NR3|PTCHD1_HUMAN    AQQKYFSN YSPVIGFYIYESIEYWN--- ---------TSVQEDVLEYT KGFVRISWFE  589

Consensus              XXXBXLXXXX XXXXXXXXXXXXXXXXXXXX XXXXXXXXXLXVXTGXXXKX XBXXXXXX  928
Q13635|PTCH1_HUMAN     YFRDWLQGLQ DAFDSDWETGKIMPNNYKNG SDDGVLAYKLLVQTGSRDKP IDISQLTK  904
Q96NR3|PTCHD1_HUMAN    SYLNYLRK-- -------------------- ---------LNVSTGLPKK- -NFTDMLR  614

Consensus              XXXXXAXXXXXX XXXXIXXXXXXXNDXVXXXA SXXXJXXXXXEXXXXXXXXX XETXXR  986
Q13635|PTCH1_HUMAN     QRLVDADGIINP SAFYIYLTAWVSNDPVAYAA SQANIRPHRPEWVHDKADYM PETRLR  962
Q96NR3|PTCHD1_HUMAN    NSFLKAPQFSHF QEDIIFS--KKYNDEVDVVA SRMFLVAKTMETNREELYDL LETLRR  670

Consensus              JXXXXXXXXXXFXX XXXXXXXXXXXXXXXXXXXX XXXNXXXXXXXXYXXXXXXX XXXX 1044
Q13635|PTCH1_HUMAN     IPAAEPIEYAQFPF YLNGLRDTSDFVEAIEKVRT ICSNYTSLGLSSYPNGYPFL FWEQ 1020
Q96NR3|PTCHD1_HUMAN    LSVTSKVKFIVF-- -------------------- ---NPSFVYMDRYASSLGAP LHNS  703

Consensus              XIXXXXXXLLFXSXXX XXXFLVXAXXLJNXWXXXXX XXXXXXXXVEXXXXGXMXLX XX 1102
Q13635|PTCH1_HUMAN     YIGLRHWLLLFISVVL ACTFLVCAVFLLNPWTAGII VMVLALMTVE--LFGMMGLI GI 1076
Q96NR3|PTCHD1_HUMAN    CI--SALFLLFFSA-- ---FLV-ADSLINVWITL-- ----TVVSVEFGVIGFMTLW KV  747

Consensus              XLXXXXVXXLIXXXXXXX XXXXXXXXXFXXXXXXXXXX XXXXALEXXXXXXLXXXXXX 1160
```

S5 Structure based sequence alignment PTCH1 and PTCHD1. ECD1 and ECD2 loops participating in ShhN binding are highlighted in turquoise and purple, respectively. Residues that participate in hydrogen bonds with ShhN in PTCH1 (6RMG) are indicated with dots. Conserved proline, identified as a key residue for PTCH1 and NPC1 function, is highlighted in yellow.
